# Emx2 and early hair cell development in the mouse inner ear

**DOI:** 10.1016/j.ydbio.2010.02.004

**Published:** 2010-04-15

**Authors:** Matthew Holley, Charlotte Rhodes, Adam Kneebone, Michel K. Herde, Michelle Fleming, Karen P. Steel

**Affiliations:** a Department of Biomedical Science, Addison Building, Western Bank, Sheffield, S10 2TN, UK; b Wellcome Trust Sanger Institute, Wellcome Trust Genome Campus, Hinxton, Cambridge, CB10 1SA, UK

**Keywords:** Emx2, Planar cell polarity, Hair cell, Inner ear, Striola

## Abstract

Emx2 is a homeodomain protein that plays a critical role in inner ear development. Homozygous null mice die at birth with a range of defects in the CNS, renal system and skeleton. The cochlea is shorter than normal with about 60% fewer auditory hair cells. It appears to lack outer hair cells and some supporting cells are either absent or fail to differentiate. Many of the hair cells differentiate in pairs and although their hair bundles develop normally their planar cell polarity is compromised. Measurements of cell polarity suggest that classic planar cell polarity molecules are not directly influenced by Emx2 and that polarity is compromised by developmental defects in the sensory precursor population or by defects in epithelial cues for cell alignment. Planar cell polarity is normal in the vestibular epithelia although polarity reversal across the striola is absent in both the utricular and saccular maculae. In contrast, cochlear hair cell polarity is disorganized. The expression domain for Bmp4 is expanded and Fgfr1 and Prox1 are expressed in fewer cells in the cochlear sensory epithelium of Emx2 null mice. We conclude that Emx2 regulates early developmental events that balance cell proliferation and differentiation in the sensory precursor population.

## Introduction

Hearing impairment affects many millions of people throughout the world (Davis, 1998). The genetic causes are diverse and involve hundreds of genes (Van Camp and Smith, 2008). Mouse mutants provide invaluable insights into human deafness and into the molecular biology of normal hearing (Brown et al., 2008; Chacon-Heszele and Chen, 2009). We have studied the homeobox transcription factor *Emx2*, whose function in inner ear development was discovered through analysis of a mouse mutant called ‘Pardon’, which was identified following *N*-ethyl-*N*-nitrosourea mutagenesis and found to have a missense mutation in *Emx2* (Rhodes et al., 2003).

*Emx1* and *Emx2* encode mammalian transcription factors homologous to the *Drosophila* empty spiracles (*ems*) head gene (Simeone et al., 1992a,b) and critical insights into their function have come from studies on null mutant mice. Mice homozygous for a null allele of *Emx2* (*Emx2*^*tm1Pgr*^, hereafter *Emx2*^*KO/KO*^) die at birth primarily as a result of severe urogenital defects (Miyamoto et al., 1997; Pellegrini et al., 1997). They also suffer specific skeletal defects, including the absence of scapulae and ilia (Pellegrini et al., 2001). The cerebral hemispheres, olfactory bulbs and hippocampus are reduced and the dentate gyrus is absent, largely due to decreased cell proliferation (Pellegrini et al., 1996; Yoshida et al., 1997). There are also defects of migration, differentiation and innervation in specific neuronal populations (Boncinelli, 1997; Cecchi, 2002; Cecchi and Boncinelli, 2000; Gulisano et al., 1996). Emx2 defines the posterior-medial area of the neocortex, influencing proliferation, identity and position of cortical neuronal progenitors (O'Leary et al., 2007; O'Leary and Sahara, 2008). Raised expression levels for Emx2 correlate with increased and decreased frequencies of symmetric and asymmetric division, respectively, in cortical precursors (Galli et al., 2002; Gangemi et al., 2001; Heins et al., 2001).

*Emx2* is expressed in amphibian (Pannese et al., 1998) and mammalian (Cecchi, 2002; Rhodes et al., 2003) otic vesicles. Heterozygous null mice (*Emx2*^*KO/+*^) suffer a minor morphological defect between the incus and malleus within the middle ear and they have a small but significantly greater number of hair cells in the apical region of the cochlea (Rhodes et al., 2003). *Emx2*^*KO/KO*^ mice lack an incus but their death at birth precludes analysis of hearing function. Pardon mutants have a missense mutation in *Emx2*. Heterozygous mutants (*Emx2*^*Pdo/+*^) closely resemble *Emx2*^*KO/+*^ mice and they suffer severe hearing impairment of 60–80 dB (Rhodes et al., 2003). Malformation of the middle ear ossicles is expected to cause a maximum conductive loss of only 40 dB so there are likely to be additional sensorineural deficits. Heterozygous pardon mutants also have about 23% more outer hair cells although the organization of the organ of Corti does not seem to be seriously disrupted. As with *Emx2*^*KO/KO*^ mice, homozygous pardon mutants die at birth and lack an incus (Rhodes et al., 2003). The severe hearing loss in heterozygous pardon mutants and the influence of *Emx2* on hair cell number suggest that examination of inner ear development in *Emx2*^*KO/KO*^ mice could provide important insights into the developmental biology of hair cells.

## Methods

### Animals and genotyping

*Emx2*^*KO/KO*^ mice were obtained from the Max-Planck-Institute, Germany on a part 129/Sv, part C57BL/6J genetic background (Pellegrini et al., 1996). They were originally generated by replacing the second and part of the third helix of the homeobox (Pellegrini et al., 1996) and were kindly provided by A. Mansouri and P. Gruss. Animal care and use were in accordance with the UK Home Office (Animal Procedures) Act 1986. Mice were killed by cervical dislocation before dissection of cochlear tissue. They were genotyped by coamplification of DNA fragments corresponding to the wildtype and mutant *Emx2* gene sequences (Lopez-Bendito et al., 2002). A single forward primer, 5′CAC AAG TCC CGA GAG TTT CCT TTT GCA CAA CG3′, was used for both wildtype and mutant sequences and two different reverse primers were used for the wildtype, 5′ACC TGA GTT TCC GTA AGA CTG AGA CTG TGA GC3′, and the mutant, 5′ACT TCC TGA CTA GGG GAG GAG TAG AAG GTG G3′, gene sequences.

### Transmission electron microscopy

Pairs of cochleae (*Emx2*^*+/+*^ *n* = 6, *Emx2*^*KO/+*^ *n* = 6, *Emx2*^*KO/KO*^ *n* = 6) were fixed in 2.5% glutaraldehyde in 0.1 M phosphate buffer (PB) for 3 h at 4 °C then washed in 10% sucrose in 0.1 M PB. Samples were post fixed in 2% osmium tetroxide, dehydrated in ethanol and embedded in Araldite resin (all reagents from Agar Scientific, UK). Ultrathin sections were cut on a Reichert Ultracut E ultramicrotome, stained with Uranyl Acetate and Lead citrate and examined using a Philips CM10 transmission electron microscope at 80 kV.

### Scanning electron microscopy

For cochlear analysis, samples (E18.5: *Emx2*^*+/+*^ *n* = 4, *Emx2*^*KO/+*^ *n* = 8, *Emx2*^*KO/KO*^ *n* = 3; P0: *Emx2*^*+/+*^ *n* = 5, *Emx2*^*KO/+*^ *n* = 11, *Emx2*^*KO/KO*^ *n* = 4) were fixed in 2.5% glutaraldehyde fixative in 0.1 M phosphate buffer then prepared using the osmium tetroxide-thiocarbohydrazide (OTOTO) method as described previously (Hunter-Duvar, 1978; Self et al., 1998). After critical point drying and gold coating, the samples were analysed using a Philips XL30 SEM at 10 kV. For hair cell counts, montages were made from the apex (80–100% of the length of the cochlear duct from the base) and the base (10–20% from the base) of the cochlea covering between 500 and 800 µm for each region of each cochlea analysed. Hair cell counts were compared by the Student's *t*-test using a significance level of *p* < 0.01. For analysis of the utricular macula, samples (E18.5: *Emx2*^*+/+*^ *n* = 4; *Emx2*^*KO/KO*^ *n* = 4) were fixed in 2.5% glutaraldehyde in 0.1 M sodium cacodylate buffer and washed with PBS before being processed with an OTOTO protocol as previously described (Hunter-Duvar 1978). After critical point drying the uncoated samples were analysed using a Hitachi FESEM 4800 operated at 5 kV, (Hitachi High-Technologies Maidenhead, Berkshire, UK). Pictures were taken at the striola, lateral to the striola and medial to the striola in the wildtypes and at similar distances from the lateral edge in the mutants.

### *In situ* hybridization and immunocytochemistry

Embryos from timed matings were dissected in cold PBS at ages ranging from E10.5 to E18.5, with E0.5 at noon the day the vaginal plug was found. At each age and for each label, at least 4 mice were used of each genotype (*Emx2*^*+/+*^, *Emx2*^*KO/+*^, *Emx2*^*KO/KO*^), except for double *Fgf8*/myosin VIIa labeling where only 4 homozygotes and 4 wildtypes were examined. For analysis of *Emx2* labeling, four 129S5 wildtype mice were used at each age. For *in situ* hybridization and immunohistochemistry on sections samples were fixed for 48 h (E10.5 embryos were fixed for 24 h) at 4 °C in 10% neutral-buffered formalin, dehydrated and embedded in paraffin. The embryos were cut into 8 µm sections and the Ventana Discovery system (Ventana Medical Systems, Inc Illkirch, France) was used for *in situ* hybridization and immunocytochemistry according to the manufacturer's instructions. A plasmid containing cDNA of *Bmp4* (Jones et al., 1991), RNA probes for *Lfng* (T3 labeled primer aattaaccctcactaaaggagCGTGTTCTTCAGGGAGTGGCAGGTC and T7 labeled primer taatacgactcactatagggagTACAGGCACACCCACTATGGGCGAC — lower case letters indicate the T3 and T7 tags) and for *Emx2* (T3 labeled primer aattaaccctcactaaaggagCTGAGAAATGTGCAGTCTGTAA and T7 labeled primer ggcgtaatacgactcactatagggCATTGACATTGACATACTTCTTGG), and antibodies against Sox2 (Abcam catalogue number ab15830 diluted 1:50), p27kip1 (Cell Signalling Technology catalogue number 2552 diluted 1:50), MyosinVIIa (Proteus catalogue number 25-6970 diluted 1:50, or 1:200 for double labeling), Jag1 (Santa Cruz catalogue number sc-6011 diluted 1:50), S100A (Abcam catalogue number ab11428 diluted 1:5000), Fgfr1 (Sigma-Aldrich catalogue number F5421 diluted 1:200), P75 (Chemicon International catalogue number AB1554 diluted 1:1000), and Prox1 (Chemicon International catalogue number AB5475 diluted 1:400) were used. The *Fgf8* plasmid used for probe preparation was kindly provided by Dr Doris Wu (National Institute for Deafness and other Communication Disorders, NIH) with permission from Gail Martin (Crossley and Martin, 1995). The double label with antibody to myosin VIIa was performed by first running the *in situ* hybridization for *Fgf8* followed by immunohistochemistry to label myosin VIIa using the Ventana Discovery System without the intervening deparaffinization and cell treatment steps. No counter stain was used to highlight tissue structure.

For neurofilament immunolabelling, wholemounts of the cochlear spiral (*Emx2*^*+/+*^ *n* = 4, *Emx2*^*KO/+*^ *n* = 4, *Emx2*^*KO/KO*^ *n* = 4) were blocked with 10% sheep serum in PBST and then incubated with anti-3A10 neurofilament antibody diluted 1:100 (Developmental Studies Hybridoma Bank). They were washed in PBST and incubated with fluorescent secondary antibody.

For whole mounts of the maculae (*Emx2*^*+/+*^ *n* = 8, *Emx2*^*KO/+*^ *n* = 24, *Emx2*^*KO/KO*^ *n* = 16) and GFAP staining of the organ of Corti (*Emx2*^*+/+*^ *n* = 3, *Emx2*^*KO/+*^ *n* = 6, *Emx2*^*KO/KO*^ *n* = 7) embryos were collected on E18.5. After bisection of heads and opening of oval window and apex of the cochlea the tissue was fixed in 4% paraformaldehyde in PBS for 2 h at room temperature. Sensory patches were dissected, permeabilized in 0.05% Triton X-100 in PBS for 30 min and kept in blocking solution (4% BSA, 2% donkey serum in PBS) at 4 °C overnight. Incubation with primary antibody (GFAP 1:200 by Sigma #G9269; β-Tubulin 1:100 by Sigma #T5201) was performed in blocking solution for 2 h at room temperature followed by 3× washing in PBS. After incubation with the corresponding fluorescent secondary antibody (donkey anti rabbit and goat anti mouse Alexa Fluor 488 1:1000 by Invitrogen #A21206, #A21052), actin was labeled with phalloidin–rhodamine (1:100 by Invitrogen #R415). Images were taken on a Zeiss LSM 510 meta confocal microscope, optimized for contrast and brightness in Adobe Photoshop CS2 and analysed using ImageJ from NIH.

To measure cochlear length the cochlear epithelium was removed, labeled with phalloidin–rhodamine, cut into 5–6 pieces and mounted on Superfrost Plus slides (Menzel, Germany). Images of the individual pieces were taken at low magnification and lengths were measured along the row of IHCs (HCs in case of *Emx2*^*KO/KO*^) with ImageJ software.

## Results

### *Emx2* in the inner ear

**Fig. 1:**
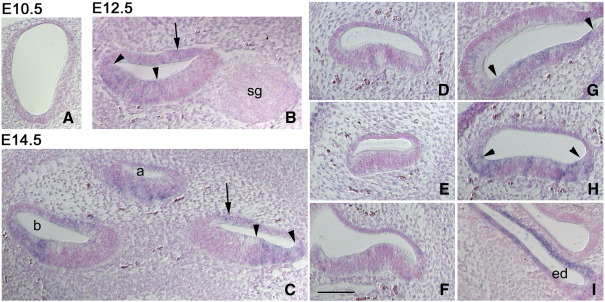
Expression of *Emx2* in the inner ear. A) Little or no expression was detected in the otocyst at E10.5. B) In the cochlear duct at E12.5 the lateral, sensory region of the cochlear epithelium was labeled (between arrowheads) and there was detectable label in the roof of the duct (arrow). No expression was observed in the spiral ganglion (sg). C) At E14.5 the expression pattern in the cochlear duct was similar to that at E12.5. The image includes sections through 3 parts of the duct from the apex (a) of the spiral to the base (b). D–F) At E14.5 there was no expression in the anterior (D), lateral (E) or posterior (F) cristae. G–H) At E14.5 Emx2 was expressed in the saccular (G) and utricular (H) maculae (defined by arrowheads). I) At E14.5 Emx2 was expressed in the endolymphatic duct (ed). Scale bar = 100 μm.

We did not detect expression of *Emx2* by *in situ* hybridization in sections of the otocyst at E10.5 (Fig. 1A). At E12.5 it was expressed in the lateral region of the cochlear epithelium in the floor and roof of the cochlear duct but not in the spiral ganglion (Fig. 1B). The labeled region in the floor of the duct included the pro-sensory cells that would give rise to hair cells and supporting cells. The endolymphatic duct (but not sac), saccular macula and utricular macula were also labeled (data not shown). At E14.5 the labeling patterns remained the same as at E12.5, with no signal recorded from the anterior, lateral or posterior cristae (Figs. 1C–I).

**Fig. 2:**
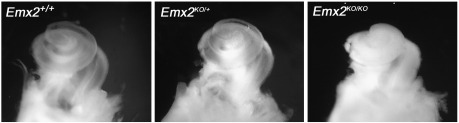
Whole dissected cochlear spirals from normal and null neonatal mice. In *Emx2*^*KO/KO*^ pups the cochlear duct was about half a turn shorter with a mean length of about 5.8 mm as opposed to 7.3–7.5 mm. There was no significant difference between *Emx2*^*+/+*^ and *Emx2*^*KO/+*^ animals at E18.5 but in *Emx2*^*KO/KO*^ animals the cochlea was significantly shorter (*p* < 0.001; the numbers of ears/embryos/litters were 8/6/3 for *Emx2*^*+/+*^, 31/21/6 for *Emx2*^*KO/+*^ and 23/15/6 for *Emx2*^*KO/KO*^).

In *Emx2*^*KO/KO*^ mice the general morphology, dorso-ventral and medial–lateral patterning of the inner ear appeared to be normal. Expression patterns of the transcription factors *Hmx3* and *Six1* at E10.5 were not detectably different to those in *Emx2*^*+/+*^ mice (data not shown). However, whilst cochleae from *Emx2*^*+/+*^ and *Emx2*^*KO/+*^ mice were 7.3 ± 0.3 mm and 7.5 ± 0.1 mm long, respectively, in *Emx2*^*KO/KO*^ mice they appeared to be more compact and measured about 5.8 ± 0.1 mm, which is approximately half a turn shorter (Fig. 2).

### Cochlear hair cells

**Fig. 3:**
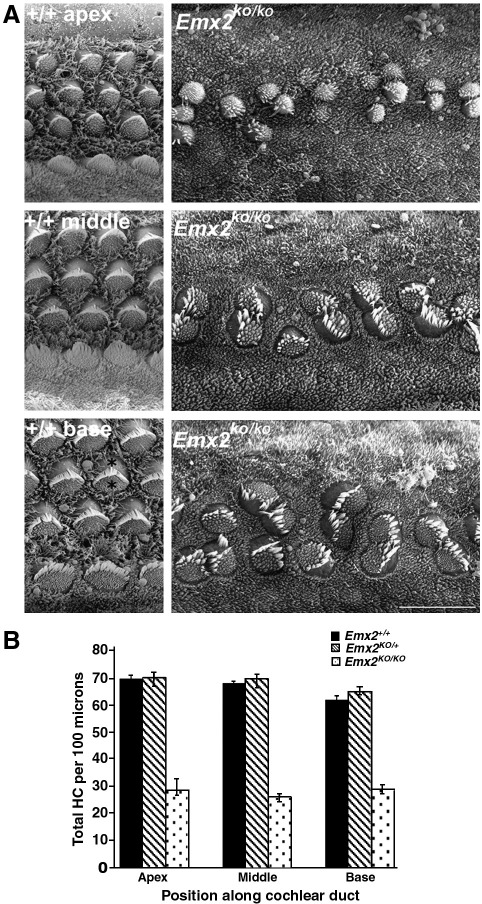
A) Scanning electron micrographs from apical, middle and basal regions of *Emx2*^*+/+*^ and *Emx2*^*KO/KO*^ neonatal mice. The bottom left panel shows 4 rows of hair cells running from left to right of the image. The 3 upper rows are composed of outer hair cells and the bottom row is composed of inner hair cells. One of the outer hair cells is marked by 2 asterisks, between which lies the V-shaped hair bundle composed of stereocilia. The cell surface inside the V is covered with microvilli whereas outside the V it is smooth. The hair bundles on the inner hair cells have a less pronounced V shape. Note that in *Emx2*^*KO/KO*^ mice there were fewer hair cells in less defined rows. Most hair cells existed singly or in pairs and lacked elements of their normal planar polarity. In morphological terms they appeared to differentiate normally if slightly slower than normal. Signs of delayed hair cell differentiation were most obvious in the apex at this stage. Scale bar = 20 μm. B) Counts of the numbers of hair cells per 100 μm of epithelium revealed no difference between *Emx2*^*+/+*^ and *Emx2*^*KO/+*^ pups but a decrease of about 55% in the *Emx2*^*KO/KO*^ pups (*t*-test — *p* < 0.001 between *Emx2*^*KO/KO*^ and both *Emx2*^*KO/+*^ and *Emx2*^*+/+*^ mice for all parts of the cochlea).

Scanning electron microscopy of the organ of Corti in *Emx2*^*KO/KO*^ pups fixed immediately after birth revealed several unusual features of the number and organization of hair cells. The first feature was that they had fewer hair cells per unit length of the organ of Corti. This was obvious from the scanning electron micrographs (Fig. 3A) from which counts revealed about 55% fewer hair cells per 100 μm (Fig. 3B). We analysed apical, middle and basal regions of the sensory epithelium separately and the relative numbers per unit length of the epithelium were similar throughout the organ of Corti. Taking the length of the cochlea into account there were some 60% fewer hair cells.

The second feature was that whereas *Emx2*^*+/+*^ and *Emx2*^*KO/+*^ pups possessed the characteristic, highly organized 3 rows of outer hair cells and single row of inner hair cells, the hair cells in *Emx2*^*KO/KO*^ pups formed only 2 poorly organized rows (Fig. 3A). However, despite the lack of such a regimented organization these rows were distributed evenly along the organ of Corti in terms of their location and density.

The third, more unusual feature was that many of the hair cells in the *Emx2*^*KO/KO*^ pups existed as very closely apposed pairs (Fig. 3A). Although pairs were most obvious there were also some single hair cells and some groups of more than 2. We counted the numbers of hair cells that were single, in pairs or in groups of up to five from the base, middle and apical regions of the cochlea in four *Emx2*^*KO/KO*^ (*n* = 1172 hair cells) and four *Emx2*^*KO/+*^ pups (*n* = 517 hair cells). All hair cells were single in the latter. In the former the results were very similar for all regions of the cochlea. About 40% of all hair cells were single and 50–55% were in pairs. Interestingly, some 6–9% were in groups of 3 and 1–2% in groups of 4 or 5 (Table 1).

A fourth feature of the *Emx2*^*KO/KO*^ pups was that there appeared to be a small but detectable delay in hair cell differentiation. Whilst cells in the basal and middle regions looked very similar to their equivalents in the *Emx2*^*KO/+*^ pups the apical cells were smaller with poorly organized hair bundles (Fig. 3A).

**Fig. 4:**
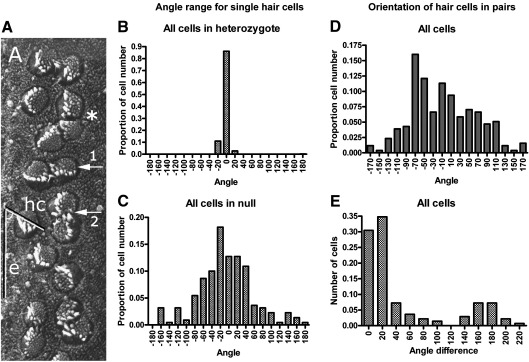
A) Scanning electron micrograph from an *Emx2*^*KO/KO*^ neonatal pup to illustrate measurements of cell orientation. Orientation was measured as the angle between the line of the hair cell bundle (hc) and the long axis of the epithelium (e). A cell with normal orientation would be at 0° to the epithelial axis. The shape of the hair bundle was not always well defined but cell orientation was further defined by the presence of microvilli on one side (arrow 1). No measurement was greater than ± 180°. Many hair cells existed in pairs facing either the same (arrow 1) or less frequently the opposite direction (arrow 2). Some cells existed in groups of 3 (*) or more. B) Range of angles for 517 hair cells recorded from apical, middle and basal regions of the cochlea in 4 different *Emx2*^*KO/+*^ pups. C) Range of angles for 1172 hair cells recorded from apical, middle and basal regions of the cochlea in 4 different *Emx2*^*KO/KO*^ pups. Despite the apparent lack of alignment most cells were orientated within a range of 180° towards one side of the epithelium. D) Range of angles for individual hair cells in pairs in *Emx2*^*KO/KO*^ pups. The distribution was similar to that of single cells. E) Range of angles between cells within pairs. Note the bimodal distribution.

The fifth feature was that the hair cells appeared to have at least partially lost their planar polarity. Their surfaces were polarized in terms of the shallow ‘V’ shape of the bundle and the presence and absence of microvilli inside and outside of the cleft of the ‘V’, respectively (Fig. 4A). Thus the cells retained intrinsic information with respect to their planar polarity. However, they appeared to have an abnormal orientation with respect to each other and to the axis of the epithelium. Since it was possible to assign a polarity to each cell it was also possible to measure the angles of hair cells relative to the epithelial axis (Fig. 4A). The orientation of cells in *Emx2*^*KO/+*^ pups was very tight with 90% of cells within a few degrees of each other and even the most deviant cells being no more than 20° either side of the transverse axis (Fig. 4B). The orientation in *Emx2*^*KO/KO*^ pups was significantly worse but not random (Fig. 4C). The mean angle and standard deviation relative to the epithelial axis was − 2 ± 10° in *Emx2*^*KO/+*^ pups and − 7 ± 63° in *Emx2*^*KO/KO*^ pups. The means were not significantly different but the variances differed substantially (*F*-test, *p* < 0.0001). Even so, 65% of measurements in *Emx2*^*KO/KO*^ pups fell within 90° of the mean and no more than a few percent of cells faced ‘backward’, suggesting that information regarding epithelial polarity was available to most cells. Paired cells shared a similar range of orientation as their single neighbours (Fig. 4D) but additional measurements of the relative angles between hair cells within pairs showed a bimodal distribution with modes at about 0 and 180° (Fig. 4E). About 65% of cells in pairs were aligned within 20° of each other but about 14% were aligned in opposite directions at 160–180°. These more precise alignments within pairs were independent of the orientation within the epithelium and they implied that paired cells shared information regarding planar polarity.

**Fig. 5:**
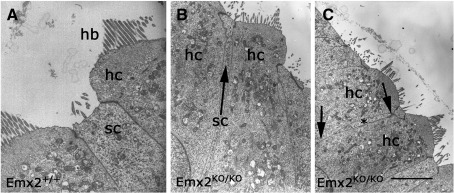
Transmission electron micrographs of sections through hair cells in *Emx2*^*+/+*^ and *Emx2*^*KO/KO*^ neonatal pups. A) In *Emx2*^*+/+*^ animals the hair cells were separated by supporting cells. B) In *Emx2*^*KO/KO*^ pups the hair cells were not spaced evenly within the sensory epithelium and were often very close to their neighbours. C) In *Emx2*^*KO/KO*^ pups some hair cells made direct contact with each other in both apical and basal regions of the cell (arrows). Part of a supporting cell is indicated by an asterisk. hc — hair cells, sc — supporting cells, hb — hair bundle. Scale bar = 5 µm.

The close association between paired hair cells raised the question of whether or not they were separated by very thin projections from the supporting cells that normally isolate adjacent hair cells. The scanning electron micrographs showed that some pairs and groups of 3 or more hair cells were so close that there would have been no space for intervening supporting cells. Although it was difficult to cut sections for transmission electron microscopy in the appropriate place and orientation this approach did provide evidence for direct contact between hair cells (Fig. 5). In *Emx2*^*KO/+*^ pups the supporting cells were obvious between hair cells whereas in *Emx2*^*KO/KO*^ pups they were often extremely slim or absent.

### Vestibular hair cells

**Fig. 6:**
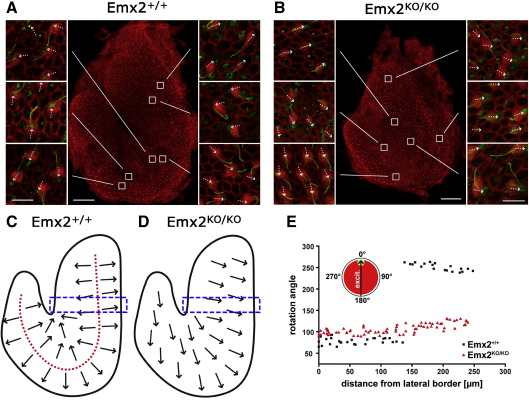
A) A surface view of the saccular macula in an *Emx2*^*+/+*^ mouse at E18.5. The central panel shows the whole epithelium with the actin labeled with rhodamine-conjugated phalloidin. The white boxes indicate adjacent panels at higher magnification. In these panels the hair cell surfaces, including the hair bundles, appear red with the kinocilia labeled with antibodies to β-tubulin in green. The dotted arrows indicate planar cell polarity with the kinocilium at the leading edge. The striola defines the line about which planar polarity is reversed (see panel C). In supporting cells the phalloidin only labeled the apical perimeter of the cells. Scale bar = 100 µm (insets = 10 µm). B) The saccular macula was smaller in *Emx2*^*KO/KO*^ at E18.5. Hair cells had differentiated hair bundles with kinocilia and stereociliar bundles. Closer examination showed that reversal of cell polarity across the striola was absent and that all hair cells faced the outer margin of the macula, as indicated by the dotted arrows in the smaller panels. Scale bar = 100 µm (insets = 10 µm). C) Diagram of hair cell orientation in an *Emx2*^*+/+*^ mouse at E18.5. The striolar boundary is shown by a dotted red line. Note that the inner edge of the horseshoe shaped epithelium is the medial side. D) Diagram of hair cell orientation in an *Emx2*^*KO/KO*^ at E18.5. The striolar boundary as defined by the reversal in cell polarity was absent. E) Measurements of the planar orientation of hair cells from analysis of the boxed regions in C and D show that cell polarity reversal was missing in *Emx2*^*KO/KO*^ mice at E18.5 even though the cells were aligned within a range of no more than 30° to the epithelial axis. Red triangles represent measurements from *Emx2*^*KO/KO*^ pups and black squares from *Emx2*^*+/+*^ pups. Note the change in polarity at the polarity reversal line about 140 μm from the lateral border only in the Emx2^+/+^ pups.

**Fig. 7:**
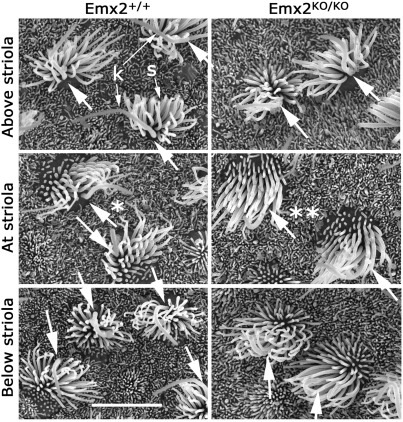
Scanning electron microscope images of hair cells from across the striola in the utricular macula at E18.5. Each image shows 2–3 hair bundles each composed of a single, long kinocilium (k) and a bundle of stereocilia (s). The stereocilia are graded in length and diameter, with the shortest ones located on the opposite side of the cell to the kinocilium. Thus cell polarity is readily assigned (large arrows). In *Emx2*^*+/+*^ mice the polarity is reversed above and below the striola and the boundary at the striola is clear (*). In *Emx2*^*KO/KO*^ mice the boundary as defined by reversal of polarity is absent (**) but polarity is uniform across the entire utricular epithelium. Scale bar = 10 µm.

The loss of alignment of cochlear hair cells with respect to the epithelium led us to examine hair cell alignment in the saccular and utricular maculae. The saccular macula is a U-shaped epithelium that is normally divided into two halves along a midline known as the striola. Either side of the striola the planar polarity of the hair cells is reversed so that the kinocilia are displaced away from it and towards the edge of the epithelium (Fig. 6A). In the saccular macula from *Emx2*^*KO/KO*^ pups the hair cells differentiated normal hair bundles but polarity reversal across the striolar region was absent (Fig. 6B). All hair cells were aligned with their kinocilia orientated to the outer (convex) edge of the epithelium at 100–120° to the lateral border. However, in contrast to the cochlea the hair cells were aligned within a range of only 30°, which was not detectably different to the range observed between hair cells within each side of the striola in normal animals (Figs. 6C–E). The surface area of the saccular macula in *Emx2*^*KO/KO*^ pups was about two thirds of that in *Emx2*^*+/+*^ pups. Similar results were observed in the utricle where hair cells in *Emx2*^*KO/KO*^ pups shared the same orientation with their kinocilia facing towards the lateral border (data not shown). The absence of the reversal in cell polarity across the striola in the utricular macula was confirmed by scanning electron microscopy (Fig. 7).

We compared anterior cristae from two *Emx2*^*+/+*^ and two *Emx2*^*KO/KO*^ pups but did not see any obvious differences between them. The kinocilia were displaced to the side of the hair cells furthest from the utricle and adjacent to the semicircular canal. A similar comparison between the lateral cristae from two *Emx2*^*+/+*^ and three *Emx2*^*KO/KO*^ pups showed that in both cases the kinocilia were displaced towards the utricle. These data were consistent with the lack of expression of Emx2 in the cristae. It is worth noting that there is normally no planar cell polarity reversal in the cristae (see Fig. 1, Fritzsch et al., 2002).

### Markers of hair cells, supporting cells and sensory epithelia in the cochlea

**Fig. 8:**
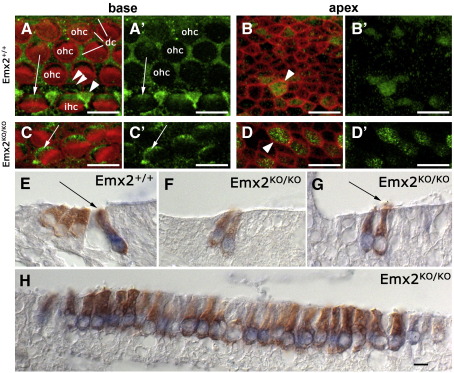
Markers for inner and outer hair cells in *Emx2*^*+/+*^ and *Emx2*^*KO/KO*^ mice at E18.5. A) Immunolabel for GFAP (green) in the basal region of the cochlea from *Emx2*^*+/+*^ mice at E18.5. Actin is labeled in red with phalloidin. The apical regions of the inner pillar cells (single arrowhead), Deiter's cells (dc) and inner hair cells (ihc and arrow) were labeled for GFAP but outer hair cells (ohc) were not. An outer pillar cell is indicated by the double arrowhead. B) As in panel A but from the apical region of the cochlea. Some cells, probably undifferentiated hair cells and supporting cells, were labeled for GFAP (arrowhead). C) As in panel A but from *Emx2*^*KO/KO*^ pups. The hair cells (arrow) were labeled but there was little sign of labeling in Deiter's or pillar cells. D) As in panel C but from the apical end of the cochlea. E) A section through the neonatal cochlea of an *Emx2*^*+/+*^ pup. All hair cells were labeled with antibody to myosin VIIa (brown) but only the inner hair cells (arrow) were labeled by *in situ* hybridization for *Fgf8* (blue nucleus). F) The two remaining rows of hair cells in the *Emx2*^*KO/KO*^ pups labeled for both myosin VIIa and for *Fgf8*. G) An occasional hair cell was negative for *Fgf8* but retained the morphology of a normal inner hair cell (arrow). H) In a section along the organ of Corti of an *Emx2*^*KO/KO*^ pup nearly all hair cells labeled for *Fgf8*. Scale bars = 10 µm.

The hair cells in the *Emx2*^*KO/KO*^ pups were not easily classified from their morphology as inner or outer hair cells. In normal animals inner hair cells and supporting cells can be labeled with antibodies against Glial Fibrillary Associated Protein (GFAP) whereas outer hair cells remain unlabeled (Figs. 8A–D). Hair cells in *Emx2*^*KO/KO*^ pups labeled for GFAP in basal and apical regions of the cochlea but the characteristic labeling pattern expected from the Deiter's cells, which normally surround the outer hair cells, and the pillar cells was virtually absent (Figs. 8A–D). A definitive label for inner hair cells is *Fgf8* (Jacques et al., 2007). In double-labeled sections of the neonatal cochlea from *Emx2*^*+/+*^ mice we identified all hair cells with antibodies to myosin VIIa and inner hair cells by *in situ* hybridization for *Fgf8* (Fig. 8E). We collected all serial sections through the cochleae and labeled every third section for Myo7a and Fgf8. We found that nearly all of the hair cells in *Emx2*^*KO/KO*^ pups labeled for both markers (Figs. 8F–H). Although the occasional hair cell was apparently unlabeled or weakly labeled for *Fgf8* this was more likely to be a false negative because the cell morphology was much closer to that of a normal inner hair cell (Fig. 8G).

**Fig. 9:**
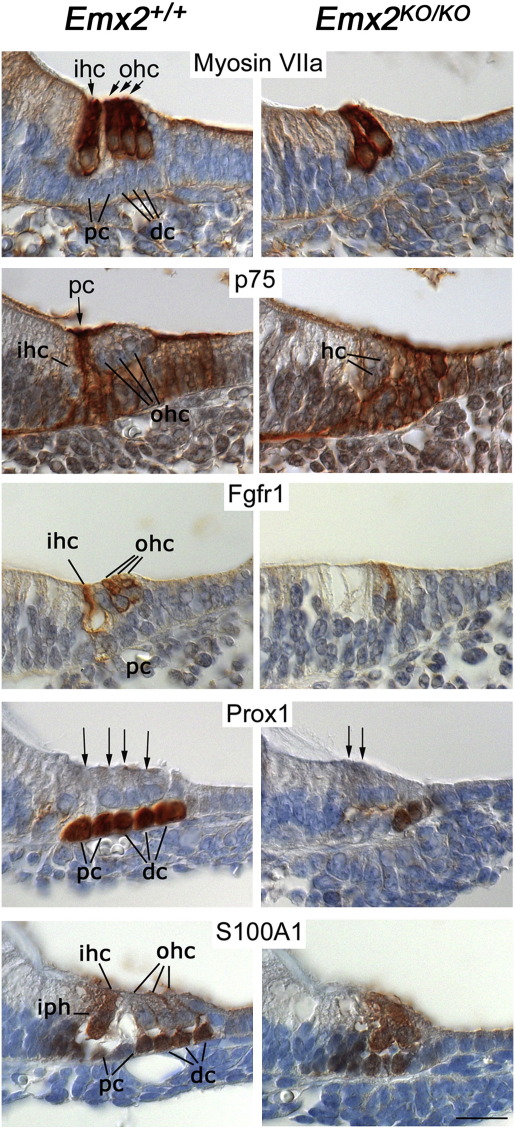
Antibody labeling for markers of hair cells and supporting cells in the organ of Corti at stage E16.5 (Myosin VIIa, p75, Fgfr1) and E18.5 (Prox1, S100A). Myosin VIIa revealed the single row of inner hair cells (ihc) and 3 rows of outer hair cells (ohc) in *Emx2*^*+/+*^ animals but only 2 rows of hair cells in *Emx2*^*KO/KO*^ animals. Pillar cells (pc), normally strongly labeled with antibodies to p75, appeared to be absent in *Emx2*^*KO/KO*^ pups. Expression of Fgfr1 was much lower in the *Emx2*^*KO/KO*^ pups, both in supporting cells and remaining hair cells. Expression of Prox1 was also much lower in *Emx2*^*KO/KO*^ pups. It was expressed only in supporting cells, specifically pillar cells and Deiter's cells (dc) at this stage, the hair cells being indicated by arrows. Antibodies to S100A labeled inner hair cells and inner phalangeal cells (iph) in *Emx2*^*+/+*^ mice but labeled the outer hair cells less strongly. In *Emx2*^*KO/KO*^ pups the boundary between inner and outer hair cells, defined by the pillar cells, was absent but the remaining hair cells were labeled strongly. Scale bar = 30 μm.

The hair cell marker myosin VIIa showed that the clear boundary that normally exists between the single row of inner hair cells and the three rows of outer hair cells was absent in *Emx2*^*KO/KO*^ pups (Fig. 9). This observation was complemented by results with the supporting cell marker, p75, which normally defines the pillar cells at this boundary. The sharp p75 labeling pattern of the pillar cells was absent in the *Emx2*^*KO/KO*^ pups although a more diffuse labeling, similar to that of the Deiter's cells, remained (Fig. 9).

The most striking changes in expression were the decreases in the fibroblast growth factor receptor 1 (Fgfr1) and the homeodomain protein Prox1. In *Emx2*^*KO/KO*^ pups the normally strong label for Fgfr1 was consistently reduced (Fig. 9) even though there was some variability in the precise labeling pattern. In *Emx2*^*+/+*^ pups the pillar cells and outer hair cells were labeled for Fgfr1 but the inner hair cells were not. In *Emx2*^*KO/KO*^ pups a few hair cells were labeled but most were not. The nuclear labeling for Prox1, which is normally observed in the two rows of pillar cells and three rows of Deiter's cells, was limited to one or two rows of supporting cells lateral to the hair cells (Fig. 9).

The calcium-binding protein S100A labeled inner hair cells, inner phalangeal cells and Deiter's cells in normal animals (Fig. 9). Interestingly, it labeled the remaining hair cells in *Emx2*^*KO/KO*^ pups, suggesting that they were equivalent to inner hair cells, a conclusion supported by the goblet-shaped morphology of the same cells labeled with antibodies to myosin VIIa as well as their GFAP and *Fgf8* labeling.

Hair cell number is regulated to a degree by notch signaling. In this context the expression of the notch ligand jagged1 might have been altered in *Emx2*^*KO/KO*^ pups. Although the sharp boundary of expression observed adjacent to the outer margin of the hair cells in *Emx2*^*+/+*^ pups was more diffuse in *Emx2*^*KO/KO*^ pups and the expression level appeared to be slightly lower, the differences were relatively small (data not shown). A similar result was observed for lunatic fringe (*Lfng*) (Fig. S1). However, at E12.5 the expression domain for *BmpP4*, which is normally expressed in cells along the lateral edge of the sensory epithelium (Morsli et al., 1998), was larger in *Emx2*^*KO/KO*^ pups and extended medially towards the *Lfng* domain (Fig. S1). The gap between these two markers was thus reduced. In all other parts of the ear their expression patterns were unchanged.

### Innervation

Given the fact that the development of numerous regions of the central nervous system is compromised in *Emx2*^*KO/KO*^ mice, we studied the innervation to the hair cells in whole mounts of individual cochlear turns from neonatal mice (Fig. S2). In *Emx2*^*KO/KO*^ pups the total number of neuronal projections appeared similar to that in controls and they terminate on the smaller numbers of hair cells available. This implies that the neuronal guidance cues within the sensory epithelium and between hair cells and nerves function effectively in the absence of Emx2. These observations are also consistent with a lack of detectable *Emx2* expression in the wildtype spiral ganglion.

## Discussion

The *Emx2*^*KO/KO*^ mouse has a remarkable phenotype that should shed light on very early developmental processes that regulate the number and pattern of hair cells in the organ of Corti. The apparently normal morphology of the hair cells demonstrates the independence of the intracellular mechanism that determines the structural polarity of the hair bundle but it also allows us to distinguish two different mechanisms that regulate the alignment of hair cells within the epithelium. The first involves information about the major axes of the epithelial tissue and the second involves direct interaction between neighbouring cells. The loss of outer hair cells, decrease in the total number of hair cells and the existence of paired hair cells suggest a subtle role for Emx2 in regulating the balance between proliferation and differentiation in hair cell progenitors. The results raise key questions regarding our current understanding of the relationship between hair cells and supporting cells during development.

### The cellular phenotype of organ of Corti in the Emx2^KO/KO^ mouse

Our results show that lack of Emx2 leads to a complete loss of outer hair cells. The remaining hair cells in *Emx2*^*KO/KO*^ mice are almost certainly inner hair cells because they not only share the same goblet-shaped morphology but also express GFAP, the calcium-binding protein S100A and Fgf8, which is a definitive marker (Jacques et al., 2007). The epithelial labeling pattern for GFAP further implies a loss of fully differentiated pillar cells and Deiter's cells (Rio et al., 2002). Definitive evidence for the absence of pillar cells comes from the lack of the characteristic labeling with antibodies to p75 (Jacques et al., 2007). The expression of the homeodomain protein Prox1 also provides evidence for the reduction in numbers of supporting cells. In normal neonatal mice it is expressed in the otocyst from E11 and down-regulated in hair cells by E16.5 when it becomes restricted to Deiter's cells and the outer pillar cell (Bermingham-McDonogh et al., 2006). The fact that it labels only a small number of cells in *Emx2*^*KO/KO*^ pups implies that there is a decrease in the overall population of supporting cells that matches the decrease in the number of hair cells.

In the frontal cortex there is a reciprocal repressive interaction between Emx2 and Fgf8 and the expression domain for *Fgf8* is bigger in *Emx2*^*KO/KO*^ mice (Cholfin and Rubenstein, 2008; Fukuchi-Shimogori and Grove, 2003; Hamasaki et al., 2004). In the normal organ of Corti approximately 25% of the hair cell population is composed of inner hair cells. Although the total number of hair cells in Emx2^KO/KO^ mice is 60% lower than normal, all of them are inner hair cells, which represent an increase of about 60% in the number of cells expressing Fgf8. Thus one might expect in *Emx2*^*KO/KO*^ mice a phenotype similar to that associated with overexpression of Fgf8. In the normal inner ear Fgf8 is expressed from about E16.5 and is restricted to inner hair cells (Jacques et al., 2007). When it is deleted from the organ of Corti, the inner and outer hair cells appear normal but the size and number of pillar cells is reduced. Ectopic expression of Fgf8 increases the expression of p75 and the number of pillar cells (Jacques et al., 2007). However, it also represses the differentiation of outer hair cells, which is consistent with an increase in *Fgf8*-expressing cells in the *Emx2*^*KO/KO*^ mice. In this respect the increase in the number of inner hair cells and associated *Fgf8* expression could partially explain the *Emx2*^*KO/KO*^ phenotype (lack of outer hair cells) although it is inconsistent with the failure of differentiation of pillar cells.

The major inner ear defects in the *Emx2*^*KO/KO*^ mice are consistent with the expression pattern for *Emx2* in normal mice. They are primarily in the lateral part of the floor of the cochlear duct, including both hair cells and supporting cells of the organ of Corti, and in the maculae. The lack of any obvious defects in the cristae correlates with the lack of expression of *Emx2*. The onset of expression, which we first detected at E12.5, also occurs shortly before the sensory progenitors exit the cell cycle and are specified as hair cells and supporting cells (Chen and Segil, 1999; Ruben, 1967).

### Planar cell polarity

Planar cell polarity (PCP) in hair cells is governed by many different genes and is disrupted in a wide range of mutant mice (Chacon-Heszele and Chen, 2009). The phenotype of *Emx2*^*KO/KO*^ mice suggests that Emx2 regulates early processes of sensory epithelial development rather than having a direct effect on the organization of PCP molecules. The hair bundles differentiated normally and despite the misalignment of hair cells in the cochlea, the hair cells in the maculae aligned well with each other (apart from the lack of polarity reversal across the striola), clearly suggesting that the basic mechanism for PCP was functional. Interestingly, the PCP proteins prickle2 and frizzled6 do not change their cellular localization across the striola (Deans et al., 2007). Mutations or deletions of PCP genes should affect all hair cells and although they are often associated with a shorter cochlear duct they do not usually include loss of hair cells or the appearance of paired hair cells (Montcouquiol et al., 2008). The complex development of the organ of Corti, including the processes of convergent extension, means that a number of developmental defects can cause hair bundle disorientation without having a direct effect on PCP molecules (Jones and Chen, 2007; Jones et al., 2008). The loss of reversal in cell polarity across the striola is one of the most interesting features of *Emx2*^*KO/KO*^ mice. In this context it is worth noting that the boundary between inner and outer hair cells is lost in the organ of Corti, which may reflect some degree of equivalence between these regions (Fritzsch et al., 2002).

The fact that hair cell polarity in the organ of Corti of *Emx2*^*KO/KO*^ pups is not random indicates that some information regarding the epithelial axis remains available to hair cells. Additional information must be transmitted locally by direct cell contact because the orientation between hair cells within pairs is more tightly regulated than that of paired or single hair cells relative to the epithelial axis. The source of information regarding the epithelial axis is unknown although it is likely to reside in the extracellular matrix and/or the supporting cells, consistent with the idea that planar polarity in mammals is a two-step process similar to that in fish neuromasts (Lopez-Schier and Hudspeth, 2006).

### Regulation of progenitors

Fgfr1 is required for proliferative expansion of auditory sensory epithelial progenitors (Pirvola et al., 2002). We have shown that expression of Fgfr1 is severely reduced in the organ of Corti in *Emx2*^*KO/KO*^ mice so this may provide some explanation for the phenotype. The outer hair cells and surrounding Deiter's cells are particularly sensitive to low levels of Fgfr1 expression (Pirvola et al., 2002). In mildly hypomorphic Fgfr1 mice outer hair cells from the outermost row are lost and in the most severe mutants only residual numbers of inner hair cells remain. A key difference is that in Fgfr1 null mice the pillar cells remain. Nevertheless, in the absence of Fgfr1 there is no obvious change in the expression of *Lfng*, *serrate1* (*jagged1*) or *Delta-like ligand 1*, which implies no direct effect on notch signaling (Pirvola et al., 2002). *Serrate1* defines the sensory patch prior to notch signaling in the chick inner ear and it is thought to be regulated by factors other than notch during the early stages of development (Daudet et al., 2007). There is no evidence for misexpression of jagged1 or of other notch signaling molecules in *Emx2*^*KO/KO*^ mice and no obvious loss of function in terms of neuronal or hair cell selection. Thus it seems that, like Fgfr1, Emx2 does not regulate notch signaling directly. However, the *Emx2*^*KO/KO*^ phenotype is not as severe as the Fgfr1 knockdown, which suggests a more complex relationship between these two genes. Despite this, our evidence suggests that outer hair cells, pillar cells and Deiter's cells fail to differentiate properly in *Emx2*^*KO/KO*^ mice and that this is caused to some extent by a failure in the expansion of the sensory precursor population. Another explanation for this failure could be the observed expansion of the expression domain of BMP4. In the chick inner ear exogenous Bmp4 reduces both the number of hair cells and the size of the sensory patches, possibly by preventing cell specification and driving the progenitors to apoptosis (Pujades et al., 2006).

### Symmetric and asymmetric cell division

The shorter cochlear duct and the smaller number of hair cells in the *Emx2*^*KO/KO*^ mouse are reminiscent of the defects due to loss of function of *Emx2* in the central nervous system. The cerebral hemispheres, olfactory bulbs and hippocampus are reduced and the dentate gyrus is absent, largely due to decreased cell proliferation (Pellegrini et al., 1996; Yoshida et al., 1997). More specifically, the existence of paired hair cells suggests a defect in the normal regulation of the balance between symmetric and asymmetric division of sensory precursors. Absolute expression levels for Emx2 correlate with the relative frequency of symmetric and asymmetric divisions in cortical progenitors (Galli et al., 2002; Gangemi et al., 2001; Heins et al., 2001). Regulation at this level can explain the observed changes in tissue size and the absolute number of specific cell types without compromising cell differentiation. For example, premature asymmetric divisions may limit expansion of the progenitor pool but allow normal cell differentiation. In its simplest form this model predicts a normal epithelial pattern in a smaller sensory epithelium. The organ of Corti is indeed smaller and there are fewer hair cells. Furthermore, the hair cells differentiate well organized hair bundles and attract what appears to be a normal level of innervation. The lack of innervation to cells other than hair cells suggests a clear distinction between cell types as expected in the normal epithelium. However, the model does not predict the observed disruption of cell patterning, especially the loss of planar polarity, or the existence of paired hair cells.

Interpretation of the model rests on the assumption that hair cells and supporting cells differentiate following asymmetric division of common progenitors. These cell types share common precursors (Fekete et al., 1998; Lawlor et al., 1999) and asymmetric division can give rise to new hair cells from supporting cells in avian and amphibian mechanosensory epithelia (Warchol and Corwin, 1996). However, during mammalian cochlear development, proliferation and differentiation are temporally separate and highly regulated. In the mouse, sensory epithelial cells destined for the apex of the cochlea pass through their final mitoses at about E12, whilst those at the base do so at about E14 (Ruben, 1967). Differentiation is then initiated in the base with a progressive delay towards the apex of about 2 days (Nishida et al., 1998). Thus basal hair cells start to differentiate soon after terminal mitosis but apical cells experience a delay of at least 4 days. Expression of the hair cell marker *Atoh1* may even be delayed as much as 8 days after terminal mitosis in apical hair cells (Matei et al., 2005). In the *Emx2*^*KO/KO*^ mouse the phenotype is consistent along the cochlea. Unless hair cells are specified in a final asymmetric division, for which there is no evidence, it seems more likely that paired hair cells in the absence of *Emx2* reflect a failure of cell specification rather than division. Nevertheless, in fish lateral line hair cells arise in pairs during development of neuromasts (Lopez-Schier et al., 2004; Rouse and Pickles, 1991) and there is direct evidence that during regeneration new hair cells are born in pairs from a single progenitor (Lopez-Schier and Hudspeth, 2006). Mammalian hair cells can divide symmetrically in the absence of either p19^ink4d^ (Chen et al., 2003) or retinoblastoma protein (Mantela et al., 2005; Sage et al., 2006) even though they do not survive. If the hair cell pairs observed in the *Emx2*^*KO/KO*^ mice are derived from a single progenitor then one would have to assume that hair cells are normally specified much earlier in the apex of the cochlea than was previously thought.

## Figures and Tables

**Table 1:** Percentage number of hair cells as singles, pairs and groups of up to five in *Emx2*^*KO/KO*^ pups.

Number of hair cells in a group	Base	Middle	Apex
1	40	38	39
2	54	55	50
3	6	6	9
4	0	0	2
5	0	1	0

## Appendix A Supplementary data

Fig. S1 *In situ* labeling for *Lfng* and *Bmp4* in animals at E12.5. *Lfng* was less intensely labeled in *Emx2*^*KO/KO*^ pups but there was no obvious change in the expression domain. The expression domain for *Bmp4* was substantially larger in *Emx2*^*KO/KO*^ pups and extended much further across the thickened part of the cochlear duct. The medial side of the epithelium is to the left. Scale bar = 100 μm.

Fig. S2 Whole mounts of apical and basal cochlear turns from *Emx2*^*+/+*^, *Emx2*^*KO/+*^ and *Emx2*^*KO/KO*^ neonatal pups labeled with antibodies against neurofilaments. In the image of the basal region of the *Emx2*^*+/+*^ animal the synaptic terminals to the inner hair cells form a single row of punctate endings (single arrow) whereas the synaptic endings on the 3 rows of outer hair cells are less conspicuous (group of 3 arrows). In the *Emx2*^*KO/KO*^ pups the innervation reflected the existence of only 2–3 rows of poorly organized hair cells. The total number of neuronal projections appears similar to that in controls and they terminate on the smaller numbers of hair cells available. This implies that the neuronal guidance cues within the sensory epithelium and between hair cells and nerves function effectively in the absence of Emx2. The synaptic endings in *Emx2*^*KO/KO*^ pups are similar to those on the inner hair cells in the normal animals.
