# Supplementary figures and images for: Emx2 and early hair cell development in the mouse inner ear
Source: Dev Biol. 2010 Apr 15;340(2):547–56. doi: 10.1016/j.ydbio.2010.02.004 (PMC2877772; doi:10.1016/j.ydbio.2010.02.004)

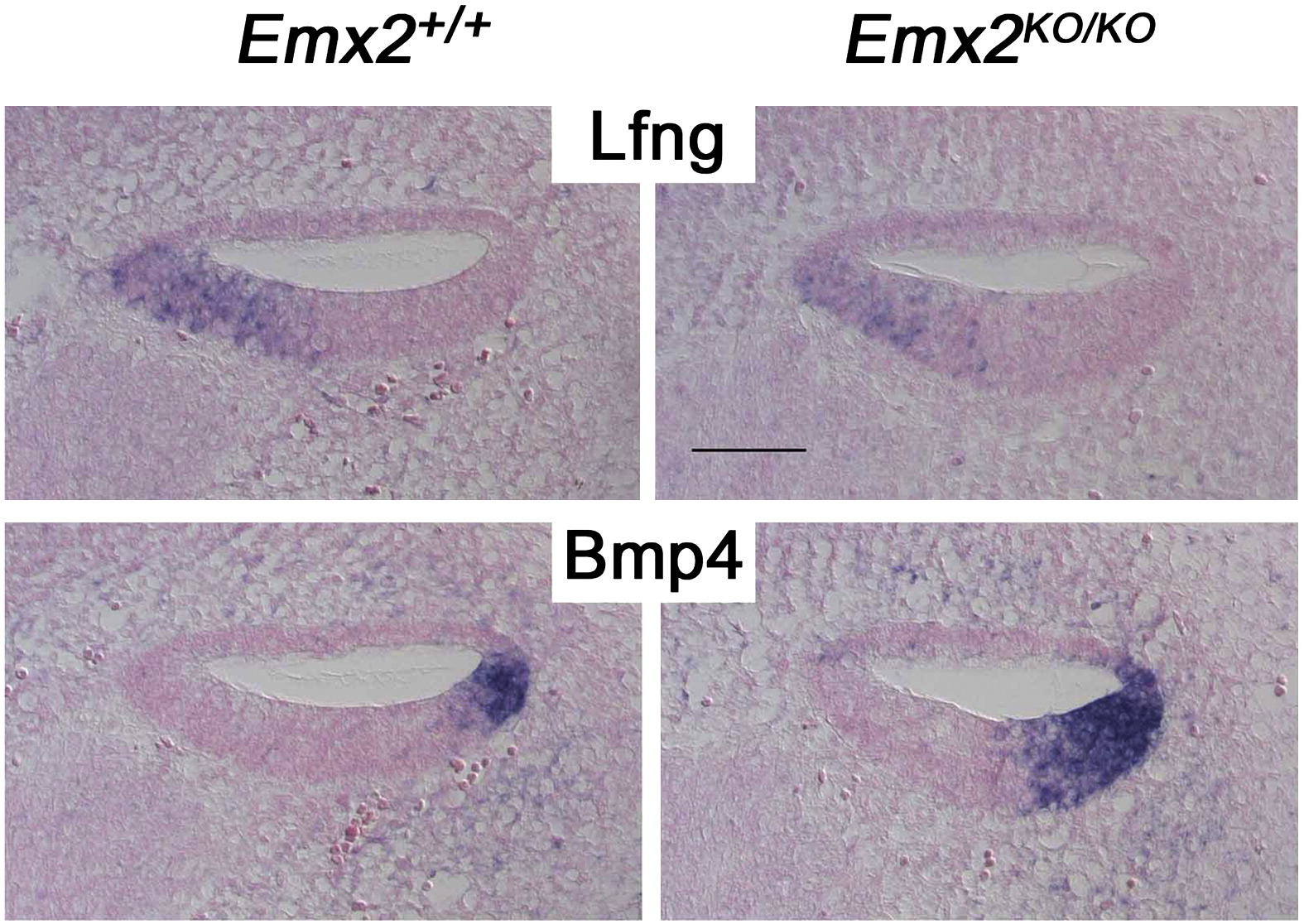

Supplement: Fig. S1 — In situ labeling for Lfng and Bmp4 in animals at E12.5. Lfng was less intensely labeled in Emx2KO/KO pups but there was no obvious change in the expression domain. The expression domain for Bmp4 was substantially larger in Emx2KO/KO pups and extended much further across the thickened part of the cochlear duct. The medial side of the epithelium is to the left. Scale bar = 100 μm. [file gr10.jpg]

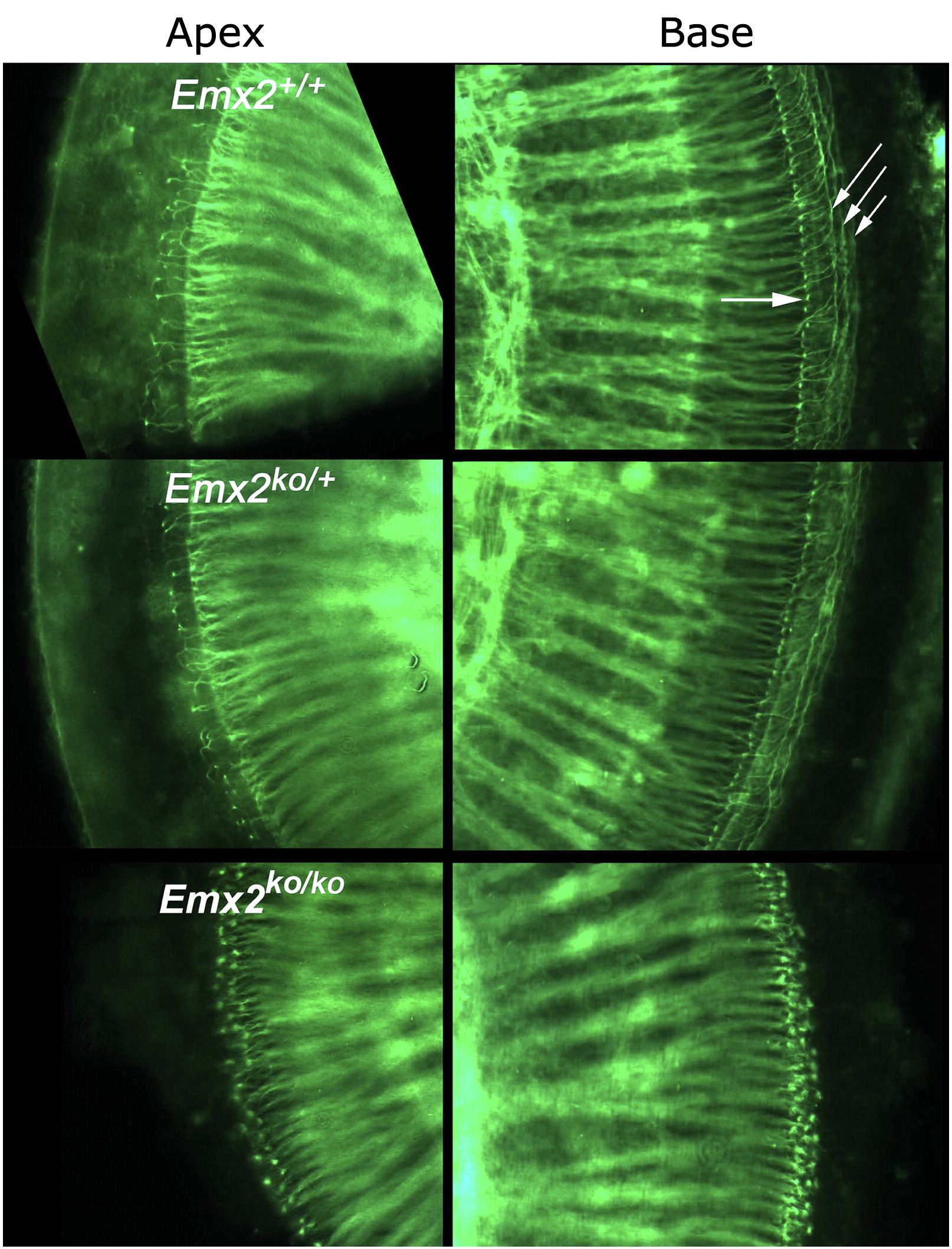

Supplement: Fig. S2 — Whole mounts of apical and basal cochlear turns from Emx2+/+, Emx2KO/+ and Emx2KO/KO neonatal pups labeled with antibodies against neurofilaments. In the image of the basal region of the Emx2+/+ animal the synaptic terminals to the inner hair cells form a single row of punctate endings (single arrow) whereas the synaptic endings on the 3 rows of outer hair cells are less conspicuous (group of 3 arrows). In the Emx2KO/KO pups the innervation reflected the existence of only 2–3 rows of poorly organized hair cells. The total number of neuronal projections appears similar to that in controls and they terminate on the smaller numbers of hair cells available. This implies that the neuronal guidance cues within the sensory epithelium and between hair cells and nerves function effectively in the absence of Emx2. The synaptic endings in Emx2KO/KO pups are similar to those on the inner hair cells in the normal animals. [file gr11.jpg]
